# Image dataset of Pune city historical places for degradation detection, classification, and restoration

**DOI:** 10.1016/j.dib.2023.109794

**Published:** 2023-11-10

**Authors:** Poonam Yogesh Pawar, Bharati Sanjay Ainapure

**Affiliations:** Department of Computer Engineering, Faculty of Science and Technology, Vishwakarma University, Survey No.2,3,4, Laxminagar, Kondhwa Budruk, Pune-411048, Maharashtra, India

**Keywords:** Degradation, Detection, Classification, Restoration, Augmentation, Deep learning, Ancient images

## Abstract

Historical and ancient murals hold clues to life in the past. This depicts the culture, worship styles, and social life of the community. This fascinates researchers and scholars who study historical bindings to showcase the modern world. Over time, most of these historical monuments have not been preserved in their original state. Most of these are affected due to natural climatic conditions, civil wars, and natural disasters. It remains clueless and imaginary about the size, shape, and color of the distorted historical monuments. This results in limitations for historical studies, archeological research, and geographical surveys. In this paper, we have studied the historical places around Pune city. Identified the locations where the monuments have been distorted and reconstructed recently. The construction age, type, color, shape, and size of the monuments are the major parameters of our study. Based on these criteria, we have captured images of these objects through different angles and camera lens. We have collected and categorized these images into folders with the names of historical places. This image dataset contains both captured and augmented images with distinct angles, scales, and directions. It also includes images captured in the daytime and evening with artificial lighting. This image dataset contains a variety of distinct image patterns that are useful as input to train computer-based supervised learning. The machine learning and deep learning algorithms perform efficiently if the input image dataset is large and distinct. Based on the predictive results generated by the machine learning and deep learning models, it is possible to virtually recreate the original monument. This would add a key value to historical research and studies.

Specifications Table

**Table:** 

Subject	Image Processing, Deep Learning
Specific subject area	*Detection of Historical Images Degradation, classification, and Restoration using Deep Learning algorithms*
Type of data	.JPEG images
How the data were acquired	Mobile device Samsung Galaxy M32 having in-built 64 MP camera used to capture the images. Some of the images captured with10X zoom lens at various Pune historical places. The images are captured at different angles and rotations distinctly.
Data format	Raw
Description of data collection	Photographs of historical objects were captured during the daytime and evening time using the rear camera of a mobile phone with different angles and directions. These images captured at timeframe from April 2023 to May 2023
Data source location	All the images were captured at Kasaba Ganapati Mandir, Lal Mahal, Shaniwar Wada, Omkareshwar Mandir, Parvati, and Ram Mandir in the city of Pune, Maharashtra, India at location 18.5204° N, 73.8567° E
Data accessibility	Repository name: Mendeley data Data identification number (doi): 10.17632/x3s9ryg5w9.2 Direct URL to data: https://data.mendeley.com/datasets/x3s9ryg5w9/2
Related research article	Pawar, Poonam, Bharati Ainapure, Mamoon Rashid, Nazir Ahmad, Aziz Alotaibi, and Sultan S. Alshamrani. 2022. “Deep Learning Approach for the Detection of Noise Type in Ancient Images” Sustainability 14, no. 18: 11786. https://doi.org/10.3390/su141811786. url: https://www.mdpi.com/2071-1050/14/18/11786

## 1 Value of the Data

• **Degradation Identification and Classification**: Over a period, ancient murals, sculptures, and paintings tends to be degraded due to natural climatic conditions. For restoration, it is essential to identify and detect the specific types of degradation. This dataset contains images with mixed sets of degradations. This will help to identify degradation and use appropriate restoration techniques.
• **Deep Learning and Machine Learning:** The modern computer-based applications use deep learning and machine learning techniques. These techniques require inputs with varying datasets to train the algorithms. The image datasets contain images captured at different angles, sizes, and augmented datasets which will help to train deep learning and machine learning models. It is possible to achieve optimized results for image restoration using this dataset.
• **Social and Historical Footprints:** Many times, because of atmospheric conditions, murals get damaged. This can lead to misleading information for society. Using this image dataset, one can predict the originality of the image with some margin. These historical footprints captured as an image dataset can be used as valuable study material for scientists and students to gain insights into history.
• **Virtual 3D Walkthrough:** Through a virtual walkthrough, one can experience a virtual visit to the original place. The images in the dataset can be bunched in sequence to build virtual 3D walkthrough applications providing real experiences of historical sites.
• **Research benefit:** The dataset contains mixed images of historical places that contribute to research areas like degradation analysis, finding degradation types, and improving the performance of restoration. Researchers can analyze and gain insights into ancient images like paintings and manuscripts. This could give more clarity about the past. This dataset could be useful for studying cultural and historical artifacts.

## 2 Objective

Historical artworks and murals are commonly affected by natural climatic conditions. This leads to degradation, fading, and cracking of the original artwork [1], [2], [3]. There are challenges to restoring murals to their originality, as it's hard to find skilled and competent craftspeople to retain the original shape. Using advanced digital image restoration techniques, it is possible to restore damaged artwork [4,5]. With the help of historical image datasets as input and optimized machine learning and deep learning algorithms, degraded artwork can be derived to match patterns of originality. By doing this, information about murals will be updated and can be stored permanently for future enhancements. Restoration of such antique artwork will significantly help researchers in historical studies and investigations [6,7]. Based on this, it will be helpful for researchers to concentrate and widen fascinating regions to preserve historical murals [8], [9], [10].

## 3 Data Description

Machine learning and deep learning algorithms require image datasets as input. A trained machine learning model identifies and categorizes the degraded images from the dataset. A good dataset must be available to train different deep learning models. The dataset includes digital images captured at historical places such as Kasaba Ganapati Mandir, Lal Mahal, Shaniwar Wada, Omkareshwar Mandir, Parvati, and Ram Mandir at Tulshibaug. The folder named “Historical Places dataset of Pune” consists of a total of 3700 augmented images. The proposed dataset consists of six folders, respectively for the above locations.

The images were captured using the Samsung Galaxy M32, which has an in-built 64 MP mobile camera. As per the needs, a camera lens is used to capture complex objects, hence the image resolution in varying sizes such as 1860 × 4032, 4624 × 2084, and 5040 × 2325. The objects are captured with high precision at various angles and directions. The augmented images also show variations in image resolution. Table 1 shows sample images and counts of original as well as augmented images, along with different subfolders.

**Table 1:** Dataset description.

Sub Folder	Sample Image	Original Images count	Augmented Images count
Kasaba Ganapati Mandir		8	140
Lal Mahal		67	864
Shaniwar Wada		324	663
Omkareshwar Mandir		145	823
Parvati		500	600
Ram Mandir		107	610
Total number of images			3700

## 4 Experimental Design, Materials and Methods

### 4.1 Experimental design

**Fig. 1:**
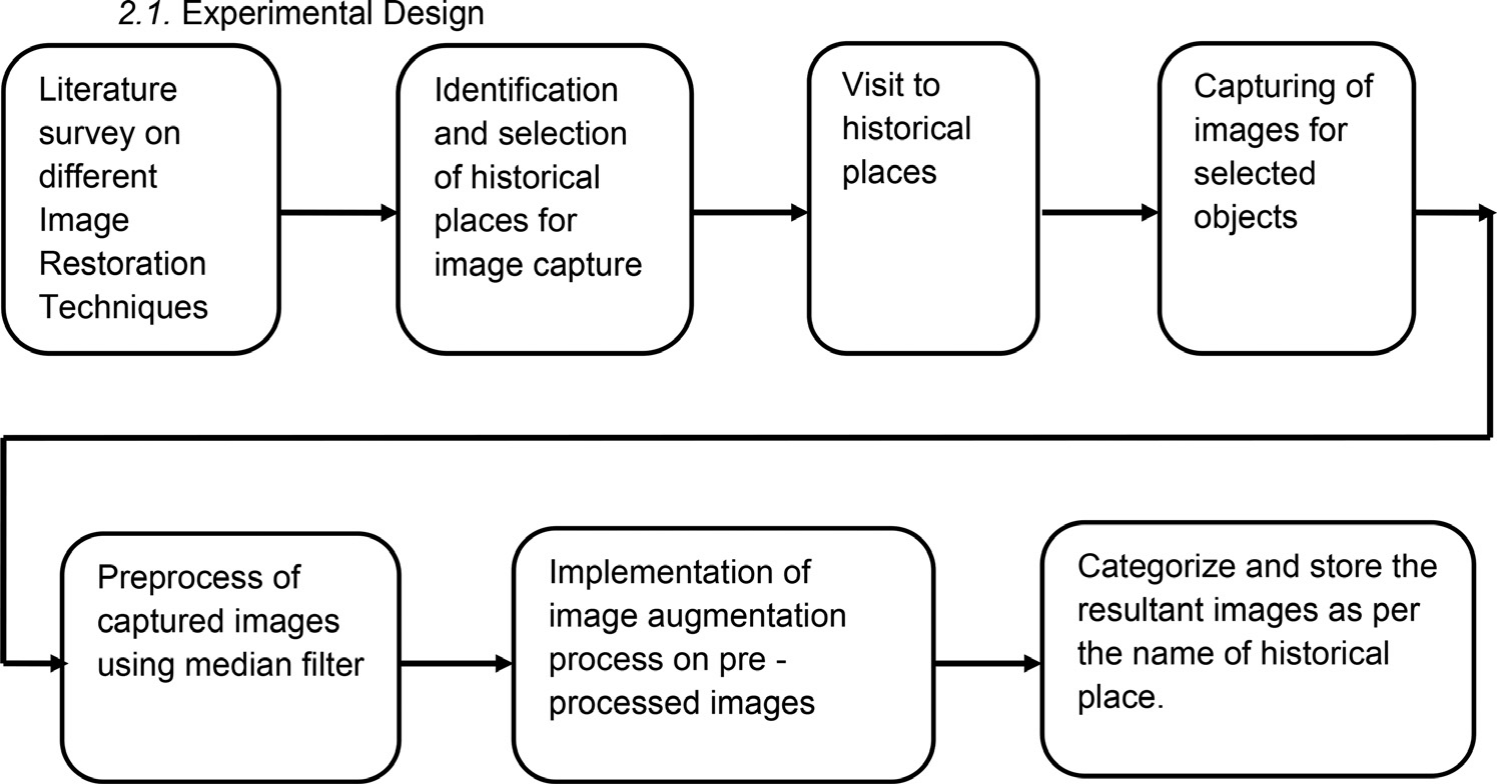
Process of dataset creation.

The images were captured during April 2023 to May 2023 around Pune Maharashtra, India, using a mobile phone camera. The process of dataset construction is shown in Fig. 1. Different locations were identified and visited, like Kasaba Ganapati Mandir, Lal Mahal, Shaniwar Wada, Omkareshwar Mandir, Parvati, and Ram Mandir at Tulshibaug, during daytime and evening time. The images are then preprocessed using the median filter to remove noise. The final dataset was prepared using an image augmentation process and categorized into six subfolders.

### 4.2 Materials

Image acquisition is done by Samsung Galaxy M32 mobile camera. All details mentioned in Table 2.

**Table 2:** Image capturing specifications.

Sr. No.	Specifications	Details
1.	Camera	a. Make and model: Samsung Galaxy M32 64 Mega Pixel b. Sensor: 64 MP c. Focus Adjustment: automatic
2.	Type of image	JPEG
3.	Camera lens used	As per needs from 1x to 10x zoom

### 4.3 Method

According to the literature survey, identified historical places and sites were visited to identify and target the objects to capture. Using image augmentation enhanced the dataset size, as machine learning and deep learning applications require large datasets. The captured images are classified so that the model is more generic, which is a well-known strategy for picture classification issues [11,12]. Rotation, scaling, different blur, different noise, and flips are applied with various settings as augmentation functions. For example, flip augmentation was performed either in the horizontal or vertical direction, and rotation augmentation was performed with an angle between 30° and 360°. The generated picture files are collected and categorized in the corresponding subfolders of the “Historical Places dataset of Pune” folder and are titled Historical place name_image number.jpg [13]. Table 3 gives details of the augmentation function to achieve the maximum image count. Different parameters could be used to get different augmented images.

**Table 3:** Image augmentation functions.

Augmentation function	Parameter Details
Flip	Horizontal/Vertical
Rotation	Degree of rotation: between 30° to 360°
Scaling	Scaling factor: 1.25 and 0.75
Bilateral Blur	Kernel size = 5
Defocus Blur	Kernel size = 7
Gaussian Blur	Kernel size = 5
Median Blur	Kernel size = 3
Motion Blur	Kernel size = 5
Gaussian Noise	Attenuation factor = 1.5
Impulse Noise	Attenuation factor = 1.25
Laplacian Noise	Attenuation factor = 1.3
Poisson Noise	Attenuation factor = 1.5

**Fig. 2:**
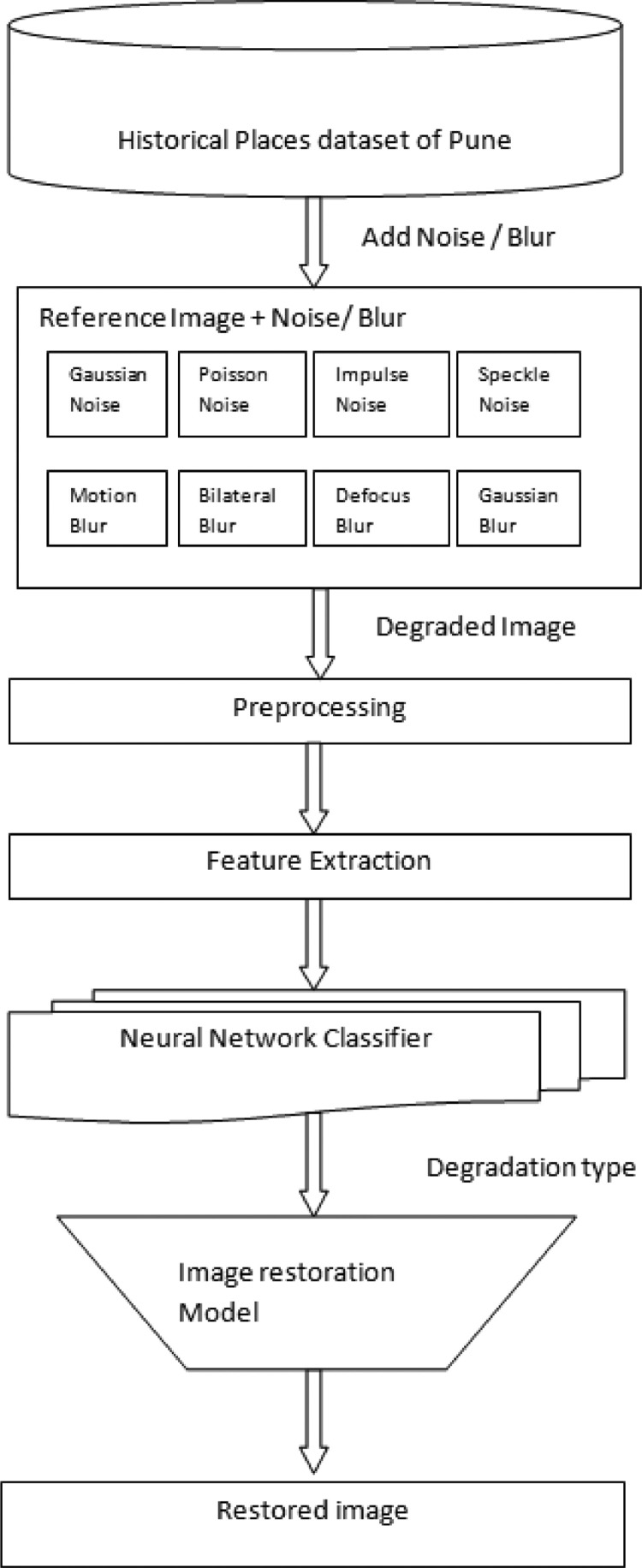
Flow for image degradation detection and restoration.

The Fig. 2 illustrates a distinctive model for image restoration using deep learning techniques. We propose supervised learning for degradation detection and restoration using the Historical Places dataset of Pune. This dataset can be used to generate degraded images for further model building. These degraded images can be input for image degradation detection through multiple levels, such as preprocessing, feature extraction, and neutral network classifiers [14]. Further image restoration model generate optimized recovered images based on model performance and accuracy. This dataset can be used in different applications such as archaeology, museums, and historical monument preservation systems, as well as the recovery of original city images after natural disasters [15].

## Ethics Statements

The work does not involve any experiments on animals and human.

## CRediT author statement

**Poonam Yogesh Pawar:** Formal analysis, Publication of the dataset, Writing the original article, Editing, Image capture & Augmentation; **Dr. Bharati Sanjay Ainapure:** Conceptualization, Data Validation, Supervision, Project administration.

## Data Availability

Historical Places dataset of Pune (Original data) (Mendeley Data)
